# Radiographic Documentation of Implant-Supported Rehabilitation Following Endodontic Failure of a Mandibular First Molar: A Case Report

**DOI:** 10.7759/cureus.112009

**Published:** 2026-07-03

**Authors:** Evelin M Mejia Ocampo, Vanessa Mora, Marcelo Romero, Maryory Perez Quevedo, Isis C De la Roche

**Affiliations:** 1 Dentistry, Windermere Center For Dentistry, Windermere, USA; 2 Dentistry, Orlando Family and Cosmetic Dentistry, Orlando, USA; 3 Dentistry, Calderon Meron Odontologia Especializada, Mérida, MEX; 4 Dentistry, Avion Dental and Orthodontics, Dallas, USA; 5 Dentistry, Holistic Dental Center, Millburn, USA

**Keywords:** dental implant, digital dentistry, endodontic failure, implant rehabilitation, implant-supported restoration, periapical pathology, periapical radiography, radiographic evaluation, ridge preservation, zirconia crown

## Abstract

Endodontic failure is a common cause of tooth loss and frequently requires extraction when persistent infection and associated pathology compromise long-term prognosis. Dental implants provide a predictable treatment option for replacing missing teeth while restoring function and esthetics. This case report describes the radiographic documentation of implant-supported rehabilitation following endodontic failure of a mandibular first molar in a 44-year-old female patient. Initial radiographic evaluation revealed a previously endodontically treated mandibular right first molar (tooth #30) associated with persistent periapical pathology and an unfavorable prognosis. The tooth was extracted, and ridge preservation was performed using a bone graft material and a barrier membrane. After a three-month healing period, implant placement was completed with radiographic verification of implant position and proximity to adjacent structures using a guide pin. Following osseointegration, restorative procedures were initiated after implant uncovering through a digital workflow utilizing an intraoral scanner, scan body, and laboratory-fabricated screw-retained zirconia crown. Sequential radiographs documented the diagnostic, surgical, and restorative phases of treatment, including extraction site preservation, implant placement, healing, and definitive restoration. The final outcome demonstrated successful implant-supported rehabilitation with satisfactory radiographic positioning and restoration of function. This case highlights the value of radiographic assessment throughout implant therapy and illustrates a predictable treatment approach for replacing a mandibular first molar affected by endodontic failure.

## Introduction

Endodontic failure remains a common challenge in clinical dentistry and may ultimately result in tooth extraction when persistent infection, recurrent pathology, or an unfavorable prognosis compromises long-term tooth retention. Although contemporary endodontic therapy demonstrates high overall success rates, treatment failure may still occur depending on case complexity, microbial persistence, procedural factors, and the duration of follow-up [1,2]. The loss of a posterior tooth can negatively affect masticatory function, occlusal stability, and overall oral health.

Following tooth extraction, dimensional changes of the alveolar ridge may complicate future implant placement [3]. Ridge preservation procedures are frequently performed to minimize post-extraction bone loss and maintain adequate bone volume for implant rehabilitation [4]. When persistent periapical pathology remains after previous endodontic treatment and the long-term prognosis for tooth retention is poor, extraction followed by implant-supported rehabilitation may represent the most predictable treatment option after consideration of endodontic retreatment and other restorative alternatives. Dental implants have become a predictable treatment modality for replacing missing teeth while restoring function and esthetics [5]. The success of implant therapy depends on proper treatment planning, implant positioning, and achievement of established success criteria [6].

Digital workflows have become increasingly integrated into implant dentistry [7]. Persistent endodontic infections, particularly those associated with microorganisms such as Enterococcus faecalis, may contribute to root canal treatment failure and persistent periapical pathology [8]. Intraoral scanning technologies facilitate accurate transfer of implant position information during restorative procedures [9]. Long-term maintenance of peri-implant health remains essential for successful treatment outcomes. Following an appropriate healing period, implant placement can provide a predictable and stable foundation for long-term restorative rehabilitation and successful functional outcomes [10].

Although implant-supported rehabilitation following endodontic failure is a well-established treatment approach, relatively few published case reports provide comprehensive sequential radiographic documentation of the entire diagnostic, surgical, and digital restorative workflow. Consequently, the educational value of this report lies in illustrating each stage of treatment, from diagnosis and ridge preservation to guide pin verification, implant placement, digital impression procedures, and definitive screw-retained restoration, thereby providing a practical step-by-step clinical reference for implant rehabilitation.

Radiographic evaluation plays a critical role throughout the diagnostic, surgical, and restorative phases of implant therapy. Sequential radiographs allow assessment of pathology, extraction site healing, implant positioning, osseointegration, and definitive restoration. This case report presents the radiographic documentation of implant-supported rehabilitation following endodontic failure of a mandibular first molar, demonstrating the progression from diagnosis through definitive implant-supported restoration.

## Case presentation

A 44-year-old healthy female presented with the chief complaint of persistent pain associated with the mandibular right first molar (tooth #30) for approximately one week following previous root canal treatment. Her medical history was noncontributory. She reported no systemic diseases, no history of previous surgical procedures, and no known drug allergies and was not taking any medications. Her last dental visit had occurred approximately one and a half years before presentation.

Clinical examination revealed tenderness to percussion associated with the previously endodontically treated mandibular right first molar. Radiographic evaluation demonstrated persistent periapical radiolucency associated with tooth #30, consistent with failure of the previous endodontic treatment and an unfavorable long-term prognosis (Figure 1). 

**Figure 1:**
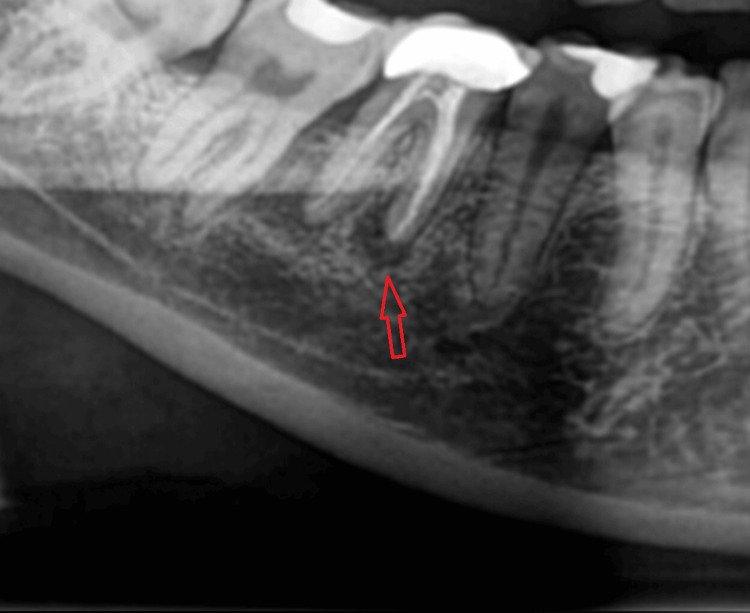
Preoperative radiographic evaluation of tooth #30 demonstrating a previously endodontically treated mandibular right first molar with persistent periapical radiolucency. Panoramic radiograph obtained at the initial presentation. The arrow indicates the periapical radiolucency associated with the previously endodontically treated mandibular right first molar (tooth #30), consistent with persistent apical pathology and an unfavorable long-term prognosis. These radiographic findings, together with the patient's clinical symptoms, supported the diagnosis of endodontic failure and the decision to proceed with extraction followed by implant-supported rehabilitation.

Following the clinical and radiographic examination, the patient was prescribed amoxicillin 500 mg every eight hours for seven days and ibuprofen 600 mg every eight hours for three days to control the odontogenic infection and associated pain before definitive treatment.

The tooth was considered to have a poor prognosis for long-term retention because of persistent periapical pathology despite previous root canal therapy. Alternative treatment options, including nonsurgical endodontic retreatment, extraction followed by implant-supported rehabilitation, and replacement with a fixed dental prosthesis, were discussed with the patient. Based on the clinical and radiographic findings, extraction followed by implant-supported rehabilitation was selected as the most predictable treatment option because it allowed replacement of the missing tooth while preserving the adjacent dentition. The risks, benefits, and available treatment alternatives were thoroughly explained, and written informed consent was obtained before treatment.

One week after the initial evaluation and completion of antibiotic therapy, atraumatic extraction of tooth #30 was performed. Ridge preservation was completed using a bovine bone graft and a resorbable collagen membrane to preserve the alveolar ridge architecture and facilitate future implant rehabilitation. A postoperative radiograph demonstrated the grafted extraction site immediately after the procedure (Figure 2).

**Figure 2:**
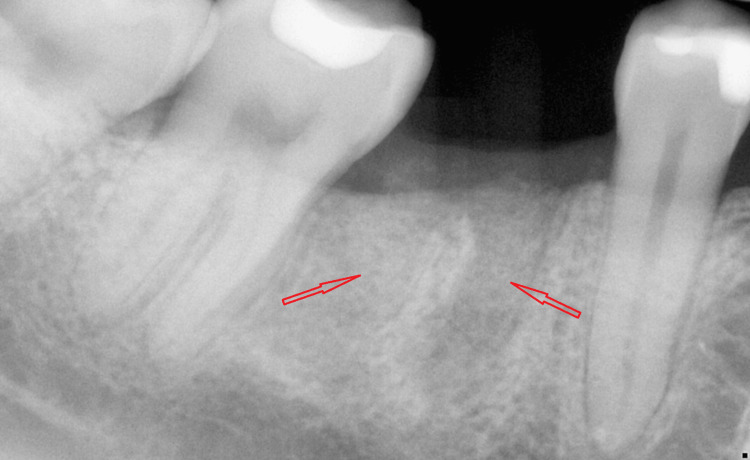
Post-extraction radiographic evaluation of site #30 following ridge preservation. Periapical radiograph obtained immediately after atraumatic extraction of tooth #30 and ridge preservation using a bovine bone graft and a resorbable collagen membrane. The arrows indicate the grafted extraction socket, demonstrating increased radiopacity consistent with the bone graft material. Ridge preservation was performed to maintain the alveolar ridge architecture and facilitate future implant placement after the planned healing period.

After a three-month healing period, implant placement was planned. A guide pin was inserted into the osteotomy site to verify implant angulation, parallelism, and the relationship to the adjacent dentition. Radiographic evaluation confirmed appropriate positioning with approximately 2 mm of clearance from the adjacent root of tooth #29 (Figure 3).

**Figure 3:**
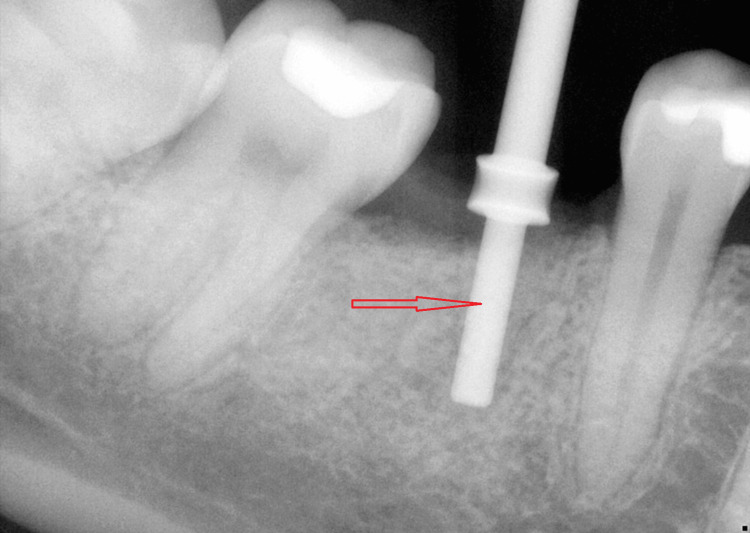
Radiographic verification of guide pin positioning three months after ridge preservation and before implant placement at site #30. Periapical radiograph obtained three months after ridge preservation during implant osteotomy preparation. The arrow indicates the guide pin positioned within the osteotomy site at tooth #30. The guide pin was used to verify implant angulation, parallelism, and the planned restorative position before implant insertion. Radiographic evaluation confirmed appropriate alignment and approximately 2 mm of clearance from the adjacent root of tooth #29, facilitating accurate implant placement.

Following confirmation of the planned implant trajectory, a BioHorizons dental implant was placed at site #30 according to the planned restorative position. Postoperative radiographic evaluation demonstrated satisfactory implant positioning within the healed ridge and appropriate spacing relative to the adjacent teeth (Figure 4).

**Figure 4:**
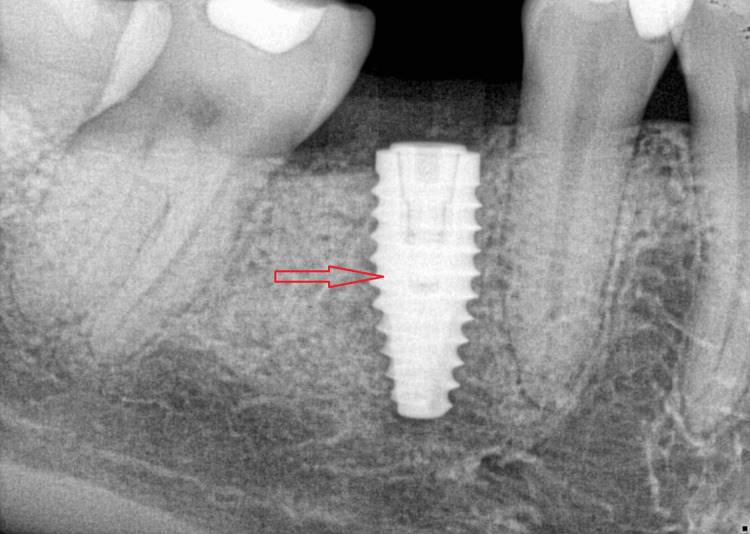
Radiographic evaluation of implant placement at site #30 following a three-month healing period after ridge preservation. Periapical radiograph obtained immediately after placement of a BioHorizons dental implant at the site of the extracted mandibular right first molar (tooth #30). The arrow indicates the implant positioned within the healed alveolar ridge. Radiographic evaluation demonstrated appropriate three-dimensional implant positioning, adequate spacing from the adjacent teeth, and favorable alignment for subsequent prosthetic rehabilitation.

Clinical evaluation demonstrated excellent primary implant stability. Resonance frequency analysis revealed an Implant Stability Quotient (ISQ) of 82, indicating excellent primary stability. A cover screw was placed, and the implant was submerged beneath the soft tissue to allow undisturbed osseointegration during healing. The clinical appearance of the implant immediately before soft tissue closure is shown in Figure 5.

**Figure 5:**
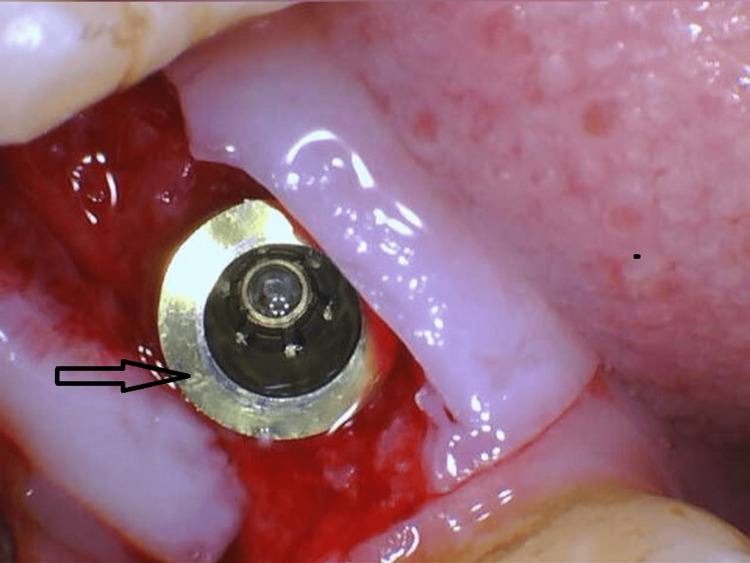
Clinical view of the dental implant immediately after implant placement at site #30, three months after ridge preservation and before surgical closure. Intraoperative clinical photograph obtained three months after ridge preservation, immediately following placement of a BioHorizons dental implant at the site of the extracted mandibular right first molar (tooth #30). The arrow indicates the implant immediately after insertion into the healed alveolar ridge. Clinical evaluation demonstrated excellent primary stability (Implant Stability Quotient = 82) before placement of the cover screw and surgical closure for submerged healing during osseointegration.

After an additional three months of healing, a second-stage surgical procedure was performed to uncover the implant and place a healing abutment to facilitate peri-implant soft tissue maturation. Following adequate soft tissue healing, a scan body was connected to the implant, and a digital impression was obtained using an iTero intraoral scanner. The digital files were transmitted to the dental laboratory for fabrication of a screw-retained zirconia implant crown (Figure 6).

**Figure 6:**
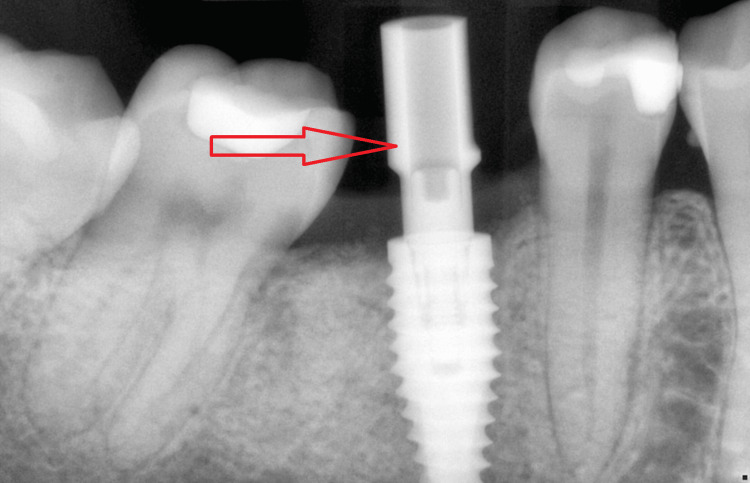
Radiographic evaluation of the scan body during the digital impression phase at site #30, six months after extraction of tooth #30. Periapical radiograph obtained six months after extraction of tooth #30 during the restorative phase of treatment. The arrow indicates the scan body connected to the implant following successful osseointegration. The scan body was used to accurately transfer the three-dimensional implant position during digital impression procedures using an intraoral scanner for fabrication of the definitive screw retained zirconia crown.

Approximately two weeks later, the definitive screw-retained zirconia crown was delivered and seated. Final radiographic evaluation demonstrated excellent adaptation of the restoration to the implant, satisfactory implant positioning, and successful rehabilitation of the mandibular right first molar (Figure 7).

**Figure 7:**
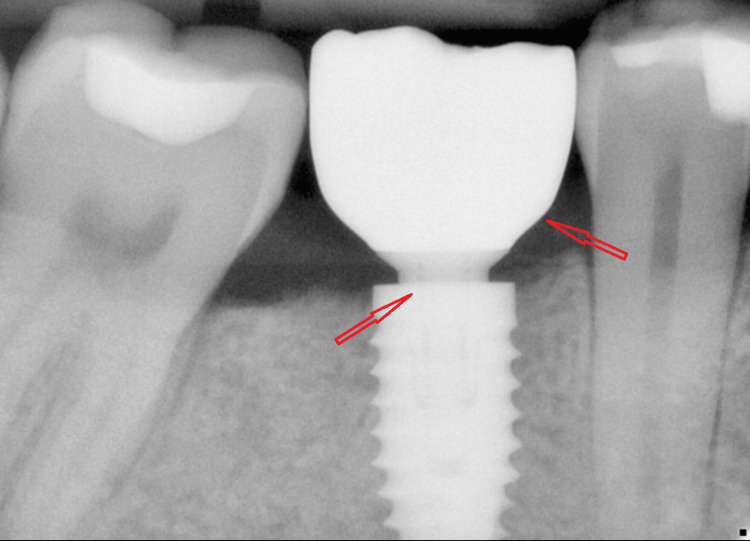
Radiographic evaluation of the definitive implant-supported crown at site #30 approximately six months after extraction of tooth #30. Periapical radiograph obtained approximately two weeks after the digital impression procedure and approximately six months after extraction of tooth #30, following delivery of the definitive screw-retained zirconia crown. The arrows indicate the definitive zirconia crown and the implant abutment interface. Radiographic evaluation demonstrated satisfactory adaptation of the definitive restoration, appropriate implant positioning, and favorable peri-implant bone support, indicating successful prosthetic rehabilitation.

Clinical follow-up examinations at three and six months demonstrated satisfactory healing, stable peri-implant soft tissues, satisfactory restoration function, and no evidence of biological or mechanical complications (Table 1).

**Table 1:** Timeline of clinical management.

Time	Clinical Event
Initial presentation	Chief complaint, clinical and radiographic examination, diagnosis of endodontic failure, amoxicillin and ibuprofen prescribed.
One week later	Atraumatic extraction of tooth #30 and ridge preservation using a bovine bone graft and resorbable collagen membrane.
Three months after extraction	Guide pin verification followed by placement of a BioHorizons dental implant. Primary stability confirmed (ISQ = 82) and submerged healing initiated.
Six months after extraction	Second-stage surgery, healing abutment placement, digital impression using a scan body and iTero scanner.
Approximately two weeks later	Delivery of the definitive screw-retained zirconia crown.
Three- and six-month follow-up	Clinical follow-up demonstrated stable peri-implant tissues, satisfactory function, and no biological or mechanical complications.

## Discussion

Endodontic failure remains a significant cause of tooth loss despite advances in endodontic therapy, including improved instrumentation, irrigation protocols, obturation techniques, and enhanced diagnostic imaging [1,2]. Nevertheless, persistent periapical pathology may still occur in some previously treated teeth and compromise their long-term prognosis. Although nonsurgical endodontic retreatment or surgical endodontic intervention may be appropriate in selected cases, extraction followed by implant-supported rehabilitation may represent the most predictable treatment option when persistent pathology and an unfavorable prognosis limit the likelihood of successful tooth preservation. In the present case, radiographic examination demonstrated persistent periapical pathology associated with a previously endodontically treated mandibular first molar, supporting the decision to extract the tooth and proceed with implant rehabilitation.

Preservation of alveolar ridge dimensions following tooth extraction is an important consideration in implant dentistry [3]. Post-extraction remodeling may reduce the volume of available bone and complicate future implant placement. Ridge preservation procedures are commonly performed to help maintain the architecture of the extraction site and facilitate subsequent implant rehabilitation [4]. In this case, satisfactory healing of the grafted extraction site allowed successful implant placement after an appropriate healing period. Implant-supported rehabilitation was selected because it provided a predictable restorative solution while preserving the adjacent natural teeth and restoring function in the edentulous area.

Accurate implant positioning is a critical factor influencing both functional and restorative outcomes [5,6]. Radiographic verification using a guide pin before implant placement confirmed implant angulation, positioning, and adequate spacing from adjacent teeth. In this case, guide pin verification confirmed approximately 2 mm of clearance from the adjacent root of tooth #29 before implant placement, providing additional confirmation of the planned surgical trajectory and facilitating optimal implant positioning for the definitive prosthetic restoration. Primary implant stability was further supported by an ISQ of 82, indicating excellent initial stability and supporting the planned conventional healing protocol. Proper implant alignment contributes to favorable prosthetic emergence, restoration design, and long-term maintenance of peri-implant tissues [7].

Digital workflows have become increasingly integrated into contemporary implant dentistry [8]. The use of intraoral scanning and scan body technology allows accurate transfer of implant position information to the dental laboratory and may improve efficiency during restorative treatment [9,10]. In the present case, digital impression procedures facilitated fabrication of the definitive screw-retained zirconia crown and contributed to accurate prosthetic rehabilitation.

The principal educational value of this case lies in its comprehensive sequential radiographic documentation of the diagnostic, surgical, and restorative phases of implant therapy. Although the individual procedures are well established, the integration of ridge preservation, guide pin verification, staged implant placement, objective assessment of primary implant stability, and a digital restorative workflow demonstrates a structured clinical approach that may serve as a practical educational reference for clinicians managing similar cases. Sequential radiographic evaluation played an essential role throughout treatment by documenting the initial pathology, extraction site healing, implant positioning, restorative procedures, and final rehabilitation. In the present case, clinical and radiographic follow-up at three and six months demonstrated stable peri-implant tissues, satisfactory restoration function, and no evidence of biological or mechanical complications. Continued long-term follow-up remains important for monitoring peri-implant health and restoration stability.

## Conclusions

This case report demonstrated successful implant-supported rehabilitation of a mandibular first molar following endodontic failure. Sequential radiographic documentation facilitated evaluation of the diagnostic, surgical, and restorative phases of treatment and confirmed satisfactory implant positioning and prosthetic rehabilitation. The staged treatment approach, which included ridge preservation, radiographic guide pin verification, objective assessment of primary implant stability, and a digital restorative workflow, contributed to a favorable clinical outcome in this patient. Clinical and radiographic follow-up at three and six months demonstrated stable peri implant tissues, satisfactory restoration function, and no evidence of biological or mechanical complications. Although limited to a single case, this report provides a practical educational reference illustrating the value of comprehensive radiographic assessment and structured treatment planning in implant-supported rehabilitation following endodontic failure.
